# Attachment strength and on-farm die-off rate of *Escherichia coli* on watermelon surfaces

**DOI:** 10.1371/journal.pone.0210115

**Published:** 2019-01-08

**Authors:** Vijay Singh Chhetri, Kathryn Fontenot, Ronald Strahan, Veerachandra K. Yemmireddy, Cameron Cason, Karuna Kharel, Achyut Adhikari

**Affiliations:** 1 School of Nutrition and Food Sciences, Louisiana State University Agricultural Center, Baton Rouge, LA, United States of America; 2 School of Plant, Environmental, and Soil Sciences, Louisiana State University Agricultural Center, Baton Rouge, LA, United States of America; University of Georgia, UNITED STATES

## Abstract

Pre-harvest contamination of produce has been a major food safety focus. Insight into the behavior of enteric pathogens on produce in pre-harvest conditions will aid in developing pre-harvest and post-harvest risk management strategies. In this study, the attachment strength (S_R_) and die-off rate of *E*. *coli* on the surface of watermelon fruits and the efficacy of aqueous chlorine treatment against strongly attached *E*. *coli* population were investigated. Watermelon seedlings were transplanted into eighteen plots. Prior to harvesting, a cocktail of generic *E*. *coli* (ATCC 23716, 25922 and 11775) was inoculated on the surface of the watermelon fruits (n = 162) and the attachment strength (*S*_*R*_) values and the daily die-off rates were examined up to 6 days by attachment assay. After 120 h, watermelon samples were treated with aqueous chlorine (150 ppm free chlorine for 3 min). The *S*_*R*_ value of the *E*. *coli* cells on watermelon surfaces significantly increased (*P*<0.05) from 0.04 to 0.99 in the first 24 h, which was primarily due to the decrease in loosely attached population, given that the population of strongly attached cells was constant. Thereafter, there was no significant change in *S*_*R*_ values, up to 120 h. The daily die-off rate of *E*. *coli* ranged from -0.12 to 1.3 log CFU/cm^2^. The chlorine treatment reduced the *E*. *coli* level by 4.2 log CFU/cm^2^ (initial level 5.6 log CFU/cm^2^) and 0.62 log CFU/cm^2^ (initial level 1.8 log CFU/cm^2^), on the watermelons that had an attachment time of 30 min and 120 h respectively. Overall, our findings revealed that the population of *E*. *coli* on watermelon surfaces declined over time in an agricultural environment. Microbial contamination during pre-harvest stages may promote the formation of strongly attached cells on the produce surfaces, which could influence the efficacy of post-harvest washing and sanitation techniques.

## Introduction

Fresh produce is highly implicated in foodborne disease outbreaks [1–3]. Produce is estimated to be responsible for 20 million illnesses, resulting in the loss of $38.6 billion annually in the US [4]. During 2010–2014, produce was on the top list of food categories responsible for *Salmonella* and shiga toxin–producing *E*. *coli* associated outbreaks [1]. Several studies have traced pre-harvest events as important causes of microbial contamination [5–8]. Produce is typically consumed raw or minimally processed. Therefore, appropriate risk management strategies should be in place in the field production stage to minimize food safety risks.

The transfer of microbial cells from sources to produce surfaces and their attachment are the key events in pre-harvest contamination. Common sources of pre-harvest contamination are soil, inadequately composted manure, irrigation water, dust, insects, an intrusion of wild animals, and human handling [9–13]. Environmental factors such as temperature, relative humidity, and contact time influence bacterial attachment and biofilm formation [14–17]. Once attached to fruit surfaces, bacterial cells initiate biofilms formation [18, 19], which is a common defensive mechanism bacteria employ to protect themselves from environmental stress [20]. Bacterial attachment is a key factor influencing the efficacy of post-harvest treatments [21, 22]. Studies indicated that chlorine and other antimicrobial treatments could become ineffective against pathogens firmly attached on produce [22–24]. Thus, an understanding of the behavior of microorganisms on produce in terms of attachment and survival is essential to develop produce safety risk management strategies.

The survival of bacterial pathogens in agricultural field settings is influenced by environmental conditions such as solar radiation, relative humidity, temperature, availability of nutrients and interaction with other natural microflora [11, 25–29]. United States Food and Drug Administration has included natural die-off as a corrective measure to reduce risks associated with agricultural water and biological soil amendments, in the Food Safety Modernization Act (FSMA) produce safety rule (PSR) [30, 31]. According to the FSMA PSR, producers that could not meet the microbial water quality criteria could use a die-off rate of 0.5 log per day while calculating the waiting period between the last irrigation and harvest. However, variable results are reported for the die-off rate of *E*. *coli*, and some studies indicated that the die-off rate could be affected by the surface characteristics of produce and the geographical location [11, 32]. Watermelons have been frequently associated with multi-state and multi-country outbreaks [33, 34]. Since watermelon grows on the ground, this produce is highly susceptible to microbial contamination during pre-harvest activities. The microbial die-off study on watermelons in the south-central part of the U.S.A may provide a commodity and geographical location specific microbial survival data for predictive die-off rate calculations. The present study examined the die-off rate and attachment behavior of *E*. *coli* on watermelon surfaces in an agricultural field setting in a south central part of the U.S.A.

## Materials and methods

### Experimental overview

A watermelon field (22 x 28 m^2^) at the Louisiana State University Agriculture Center (LSU AgCenter) Botanic Gardens in Baton Rouge, Louisiana, in summer 2016 was used in this study. Watermelon variety ‘Legacy’was sourced from Reimer Seeds (MD, USA), and planted into jumbo 6-0-6 plastic inserts and trays. The medium used was Sunshine Mix 1. The medium, inserts, and trays were purchased from BWI Companies Inc. (Nash, TX). To achieve uniform germination, seeds planted into the trays were placed on heat mats set at 29°C for 24 hours. The trays were removed off the mats but kept in the greenhouse for 3 weeks. After the first true leaf emerged, seedlings were fertilized with Peter’s 20-20-20 (ICL Specialty Fertilizers, the Netherlands) weekly at 200 ppm N. Five hundred pounds (65 lbs actual N per acre rate) of Expert Gardener 13-13-13 (Schultz Company St. Louis, MO) was incorporated into the field 7 days prior to planting. After transferring the plants to the field, overhead irrigation was used to establish plants, until the vines began to run. Irrigation was not applied within the last month of growth prior to the harvest. In this study, only municipal water was used for irrigation, and no biological soil amendments of animal origin were added to the seedlings or to the field thus minimizing microbial contamination.

### Testing natural coliform and *E*. *coli* levels on the watermelon surfaces

After 80 days, watermelon samples (n = 25) were randomly harvested from different locations of the field using sterile disposable gloves. Watermelon length, width (L x W cm^2^) and fresh weight (g) were measured. The visible soil was removed using sterile tissue paper. Each watermelon was cut horizontally into halves, upper (facing to sunlight) and lower (facing to the ground) and the edible portion was removed using sterile stainless-steel knives. The area of each watermelon surfaces was measured (Area = π * Semi-Major Axis * Semi-Minor Axis). The areas of the half watermelon surfaces ranged from 187 to 366 cm^2^. Watermelon rinds from each halves were then aseptically transferred into a sterile polythene bags and immediately transported to the laboratory maintaining 4ºC. After receiving the samples in the lab, 0.1% peptone water (100 mL) was added into each bag and the rind was hand massaged with peptone water for 5 min to dislodge the microorganisms from the melon surfaces. The supernatant after massaging was used for the microbial analysis. Petrifilm™ EC plates (3M^™^ Microbiology Products Co., St. Paul, MN) were used to enumerate coliform and *E*. *coli* levels on the watermelon surfaces.

### *E*. *coli* inoculum preparation

This study used a cocktail of three *E*. *coli* strains (ATTC 23716, 25922 & 11775). These strains are among the few well-characterized potential surrogates for *E*. *coli* O157:H7 for use in field trials [35, 36]. Frozen cultures were activated in three successive passes and harvested to an initial inoculum size of 7–8 log CFU/mL by following the procedure described by Adhikari et al (2016) [37]

### Curli expression of *E*. *coli*

The level of curli expression of *E*. *coli* strains was determined using the congo red binding assay [38, 39]. Each culture was streaked on Congo red indicator (CRI) agar (0·1% tryptone, 0·05% yeast extract, 1·5% agar, 0·004% Congo red and 0·002% Coomassie brilliant blue) and incubated at 22°C and 32°C for 48 h. We recorded *E*. *coli* producing red colonies as curli producers, while those producing colorless colonies were recorded as curli negative.

### Watermelon inoculation with *E*. *coli*

Watermelons (n = 160) in the field were selected at random, and a circle (50 cm^2^) was drawn on the upper surface of each watermelon fruits using a permanent marker pen. Prior to inoculation, the *E*. *coli* cocktail was agitated 25 times in a 30 cm arc to ensure thorough mixing. Each marked surface was then spot inoculated with 200 μL inoculum distributed into 15 small droplets and spread using a sterilized cotton swab.

### Recovery of strongly and loosely attached *E*. *coli* from watermelon surfaces

At 24 h time intervals (0, 24, 48, 72, 96 & 120 h), six watermelon samples were harvested randomly each time (n = 36). The inoculated rind area of each watermelon was cut as a disk (50 cm^2^, 42 g) and separated from the edible portion using a sterile stainless steel knife. We placed the disks in sterilized plastic bags and transported to the laboratory maintaining 4°C. After that, we separated the outer green peel (8 g) using a sterilized vegetable peeler and placed in a sterile Security-Snap Bottle (Fisher Scientific, USA). The attachment assay was performed using the method described by Ells and Hansen (2006) [15] with a slight modification. Briefly, 25 ml of PBS (pH 7.2) containing 0.1% Tween 20 was added to the peel and vortexed for 20 s to remove loosely attached bacterial cells. We repeated the washing process in a new bottle with fresh PBS containing 0.1% Tween 20. We collected and mixed spent wash solutions for the enumeration of loosely attached *E*. *coli* cells. The washed rinds were transferred to 50 ml Falcon® tubes containing 25 mL of PBS and homogenized for 30 s at high speed using a Fisher Scientific™ 150 Hand Held Homogenizer (Fisher Scientific, USA). Between each sample, we sanitized the homogenizer with 70% ethanol and rinsed three times with sterile distilled water to remove residual alcohol. The homogenate was used to enumerate strongly attached *E*. *coli*. Enumeration of *E*. *coli* was done by plating the homogenates and their respective wash solutions on 3M™ Petrifilm *E*. *coli*/Coliform count plates and incubating the plates at 37°C for 48 h. The attachment strength (S_R_ value) was calculated as the ratio of bacterial population recovered from homogenate (strongly attached cells) to the total bacterial cells (strongly attached + loosely attached) [14]. The weather data for the study period was retrieved from https://www.wunderground.com.

### Scanning electron microscopy analysis

Watermelon rinds obtained after double PBS-tween 20 buffer wash were cut off (about 2x2 mm area and 0.5 mm thickness) using a sterile blade. The cut pieces were fixed overnight in an FAA solution (95% Ethanol (50 mL), Glacial Acetic Acid (5 mL), 40% Formaldehyde (10 mL), distilled water (35 mL). The fixed samples were dehydrated in a graded ethanol series (50%, 70%, and 100%). The samples were then dried in a Denton DCP Critical Point Dryer (Denton Vacuum, LLC, USA) with CO_2_ as the transition gas. We mounted the samples on aluminum SEM stubs and coated with platinum in an EMS 550X sputter coater (Electron Microscopy Sciences, USA). The samples were examined with a JSM-6610 scanning electron microscope (JEOL USA Inc., USA) at an accelerating voltage of 10 KV in the high vacuum mode.

### Chlorine treatment

Watermelons (n = 18, 9 each at 30 min and 120 h post-inoculation) were harvested using sterilized disposable gloves. Each watermelon was completely dipped into 10 liters of aqueous 150 ppm chlorine (available) solution (25°C, pH 7.3) (Clorox®, Oakland, CA, USA) for 3 min. Chlorine concentration was measured by Clorox Smart Strips (Clorox®, Oakland, CA, USA). After the treatment, we removed the inoculated disks from the fruits as previously mentioned, and separated the outer green peel using a vegetable peeler. The peel was homogenized in 100 mL of Dey-Engley (D/E) Neutralizing Broth using a Fisher Scientific™ 150 Hand Held Homogenizer (Fisher Scientific, USA). We enumerated *E*. *coli* populations in the homogenate using 3M™ Petrifilm *E*. *coli*/Coliform count plates.

### Statistical analysis

Coliforms and generic *E*. *coli* populations recovered from the watermelon samples by direct plating on the 3M petrifilms were converted to log CFU/cm^2^. The bacterial die-off and attachment strength over the time was analyzed using ANOVA with Proc mixed feature of SAS 9.4 (SAS Institute, Cary, NC, USA.). The level of statistical significance was p<0.05 in all cases.

## Results

### Levels of natural coliforms and *E*. *coli* on upper and lower half surfaces of watermelons

All the tested samples were positive for coliforms. The coliforms levels were not significantly different (P>0.05) with an average population of 2.20±0.18 log CFU/cm^2^ and 2.65±0.17 log CFU/cm^2^ on the upper and the lower half samples, respectively. We observed a low prevalence of natural *E*. *coli* on the surfaces of the watermelon fruits (S1 Appendix). Out of 25 watermelons, 10 were positive for *E*. *coli* with five upper half and six lower half positive samples. Only five watermelons had *E*. *coli* levels more than 1 log CFU/cm^2^. Although some lower half surfaces had higher count (up to 2.64 log CFU/cm^2^) compared to upper half surfaces (< 1 log CFU/cm^2^), overall *E*.*coli* prevalence or level was not significantly different between the surfaces.

### Die-off rate and attachment strength of *E*. *coli* on watermelon surfaces

**Fig 1:**
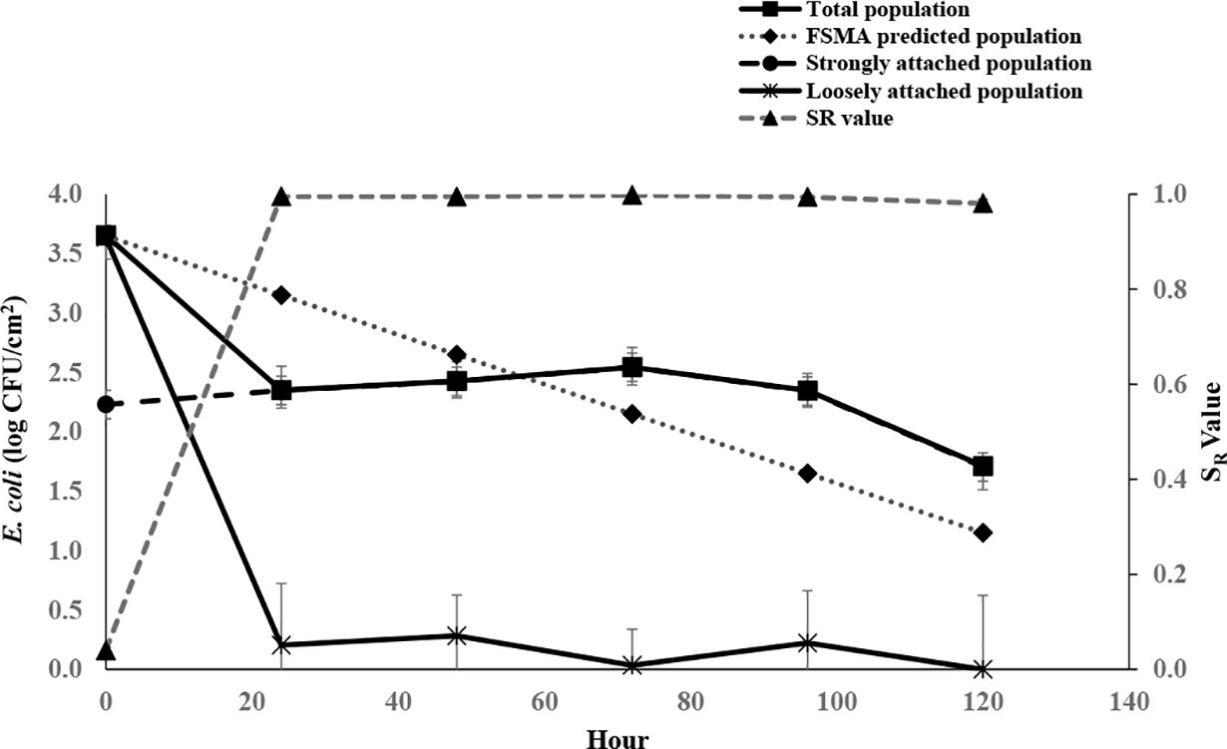
Die-off and attachment of *E*. *coli* on watermelon surfaces. Average *E*.*coli* count on the watermelon surfaces and FSMA- PSR predicted count on produce surfaces based on the die-off rate of 0.5 log CFU/day, up to 120 h (Day 6). The population of loosely attached and strongly attached cells is also shown for up to 120 h. S_R_ Value (Attachment Strength) = Strongly Attached Cells/ Total Cells (strongly attached+ loosely attached). Data are shown as mean values ± standard deviation.

The die-off rate of *E*. *coli* inoculated on the watermelon surfaces is shown in Fig 1. A significant reduction (P<0.05) in *E*. *coli* population (total) was observed within 24 hours (Day 2). In the period between 24 to 96 h, there was no significant (P>0.05) reduction in *E*. *coli* levels. At 120 h, the total reduction was 1.94 log CFU/cm^2^ (from 3.65 log CFU/cm^2^ to 1.71 log CFU/cm^2^). The daily die-off rate was variable during the study period, and the daily die-off pattern was more likely multiphasic up to 120 h. The highest daily die-off was 1.30 log CFU/cm^2^ in day 2 (after 24 h) followed by 0.63 log CFU/cm^2^ in day 6, which were greater than the FSMA produce safety rule predicted die-off rate (0.5 log CFU/day) on produce surfaces. However, in other days, the die-off rates were lower than the predicted value.

All the tested *E*. *coli* strains (ATCC 23716, ATCC 25922 and 11775) produced intense red colored colonies on CRI agar at 32°C indicating a strong curli expression. However, at 22°C, only *E*. *coli* ATCC 25922 showed curli expression. Other tested strains (ATTC 23716 and 11775) produced curli negative (colorless) colonies at that temperature.

The level of *E*. *coli* attachment on the watermelon surfaces is shown in Fig 1. The attachment strength (S_R_) of *E*. *coli* on the watermelon surfaces increased significantly (P<0.05) within 24 h. The initial *S*_*R*_ value at 0 h (i.e., after 30 min of inoculation) was 0.04, which increased to 0.99 after 24 h, and this level maintained up to 120 h. There was a significant reduction (P<0.05) in loosely attached population (from 3.6 log CFU/cm^2^ to 0.20 log CFU/cm^2^) in 24 h. Afterward, the lower level of loosely attached population maintained up to 120 h. However, there was no significant change in the strongly attached population up to five days (the graph overlapped by total count). Thus, the increase in S_R_ over time was mainly attributed to the decrease in the loosely attached population, given that the population of strongly attached cells was constant.

### Scanning electron microscopy (SEM) analysis

**Fig 2:**
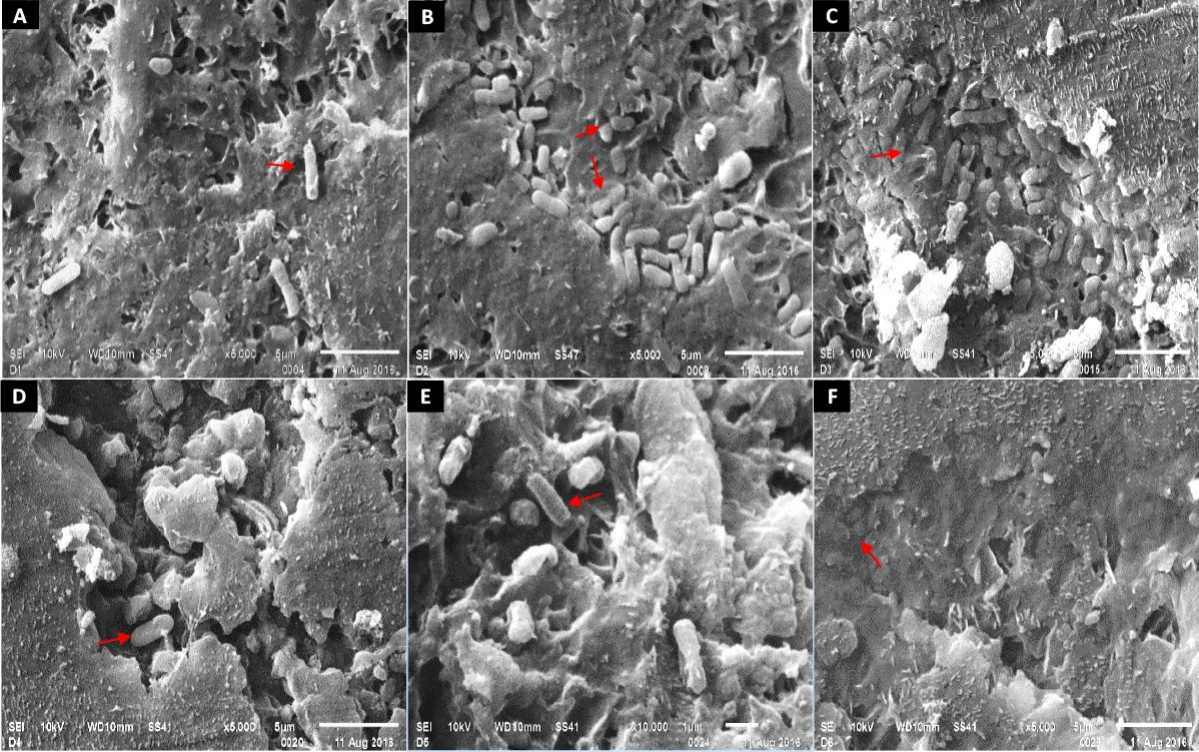
Scanning electron micrographs of *E*. *coli* inoculated watermelons surfaces. SEM images (SEI 10 KV, 5,000x & 10,000x) of watermelon surfaces after inoculating with *E*. *coli* and leaving them in an agriculture field for 0 h (A), 24 h (B), 48 h (C), 72 h (D), 96 h (E) and 120 h (F).

The SEM micrographs showed a strong colonization of small rod-shaped bacterial cells on the surface of watermelons after 24 h (Fig 2). Since these samples were inoculated with *E*. *coli* cells (~ 4 log/cm^2^) and there was a trend of attachment over time, the majority of those cells were possibly *E*. *coli*. At 0 h, individual cells were predominant on the watermelon surfaces (Fig 2A). After 24 h, the cells were smaller and clustered together. As time progressed, the cells became overlaid with a sheath of substances resembling an extracellular matrix (Fig 2C). After 120 h, it was hard to observe bacterial cells on the watermelon surfaces.

### Efficacy of chlorine treatment

**Fig 3:**
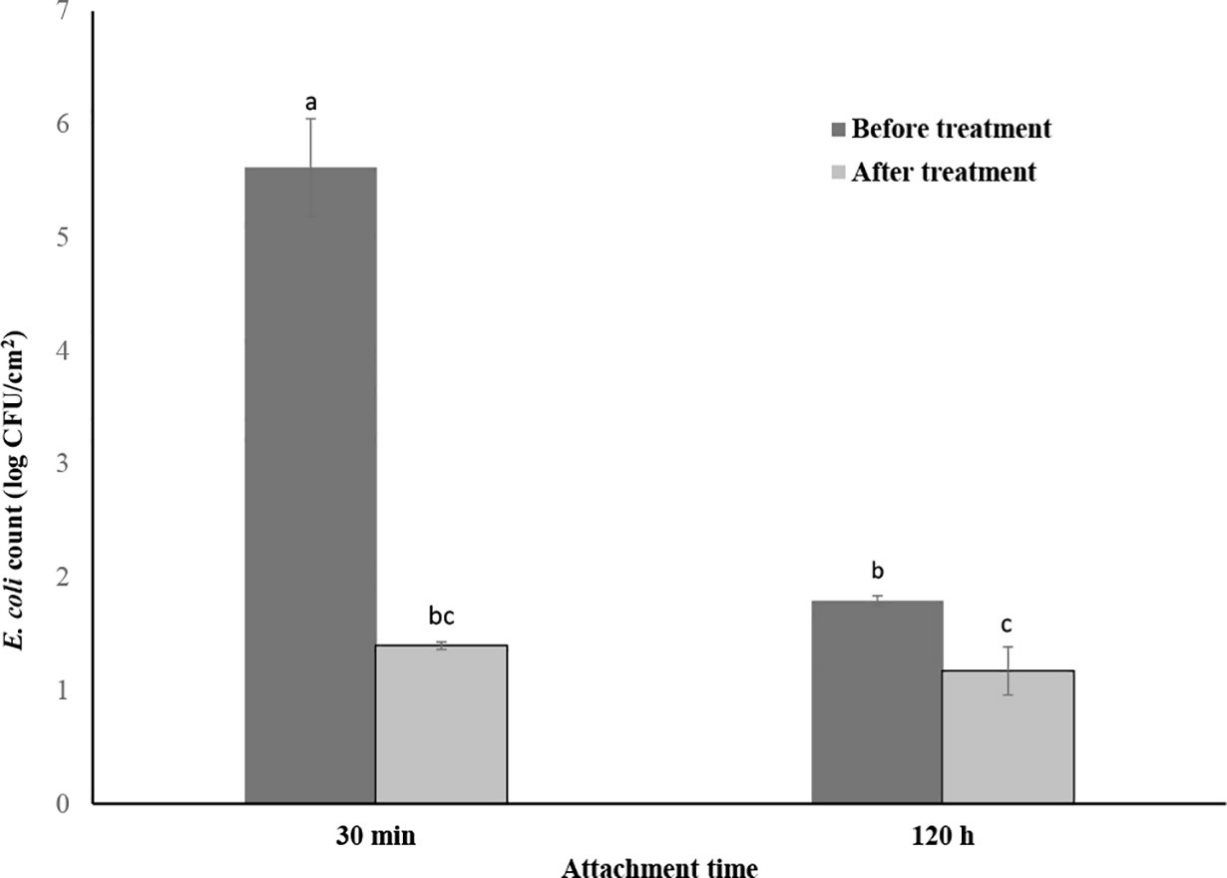
Efficacy of chlorine (150 ppm for 3 min) on *E*. *coli* inoculated watermelon surfaces. 30 min: Inoculated 30 min prior to the treatment; 120 h: Inoculated 120 h prior to the treatment. The bar diagrams with different lowercase letters on the top are significantly different (P < 0.05) from each other. The detection limit of the test was 0.28 log CFU/cm^2^.

The efficacy of aqueous chlorine (150 ppm) against *E*. *coli* on the watermelon surfaces at two different attachment times (30 min and 120 h) is shown in Fig 3. The efficacy of chlorine treatment in reducing the *E*. *coli* level was significantly (P< 0.05) higher on the freshly inoculated watermelons than on those watermelons inoculated 120 h before the treatment. The average reduction due to the chlorine treatment was 4.2 log CFU/cm^2^ and 0.62 log CFU/cm^2^ on 30 min and 120 h post-inoculation watermelon samples, respectively.

## Discussion

### Coliforms and *E*. *coli* were uniformly distributed on the watermelon surfaces

The lower part of watermelon surfaces which sits on soil is generally assumed to have a higher microbial level than the upper part of the watermelon. Studies on melon-rinds indicated a higher level of microbial contamination on ground spots as compared to the non-ground spot areas of melon rinds [40, 41]. In addition, the upper surfaces may have a greater chance of exposure to sunlight than the lower surfaces. However, we did not observe a significant difference in the levels of coliforms between upper and lower half surfaces of the watermelon. This result agrees with our previous study, which observed a similar level of coliform and generic *E*. *coli* counts on watermelons with different levels of sunlight exposure caused by the surrounding vegetation [42]. The top part of watermelon surfaces may be equally prone to the bacterial contamination as the bottom part in the agriculture field. Results were observed in the condition where municipal water was used for irrigation without the addition of biological soil amendments of animal origin. Therefore, further investigation to establish an association between the distribution of microbial cells on produce surfaces and sources of contamination is needed.

### Majority of *E*. *coli* die-off was within 24 h of contamination

Our study demonstrated that die-off of *E*. *coli* does occur on watermelon rind surfaces in an agricultural field environment. The reduction in culturable *E*. *coli* population on the surface of watermelon fruits was significant at 24 h. However, the reduction rate did not follow the same pattern over time. The rapid decline in *E*. *coli* population in the first 24 h was evident in most of the studies [43], which was likely due to the sudden change in the environmental conditions. Limitation of nutrients, sunlight, desiccation, and competition with surface microflora might have been the predominant stress factors [43]. Several studies have reported an average die-off rate of around half a log on produce matrices, such as lettuce and spinach [44, 45]. For instance, Weller et al (2017) [46] demonstrated a mean *E*. *coli* die-off rate of 0.52 log MPN on lettuce heads under field condition in Freeville, New York. Similarly, Barker-Reid et al (2009) [44] observed a total reduction of *E*. *coli* level on lettuce by 2.2-log in 5 days (the average daily die-off rate of 0.44 log) in southeastern Australia. Wood et al (2010) [45] observed an exponential decline in *E*. *coli* population on spinach after irrigation over the time under field condition in Canada. In contrast, we did not observe any significant change in the *E*. *coli* levels between 48 to 96 h. This result may be attributed to the bacterial ability to adapt to a new environment and/or to develop resistance to the environmental stresses, by altering their cellular physiology [47–51]. However, further studies are needed to better understand the survival mechanisms of bacterial cells, specifically on the surface of watermelon fruits.

We compared the daily die-off rate with the rate recommended by FSMA produce safety rule (0.5 log per day) to see if it followed a similar pattern. Out of 6 days, two days (day 2 and day 6) had higher daily die-off rate than the recommended rate. However, for other days the population did not change significantly. In other studies, daily die-off rate of *E*. *coli* on produce varied from 0.4 to 1.64 log MPN/day [26, 46, 52, 53]. As the geographical locations where the studies were conducted were different among studies, the variation in die-off rate might be due to the differences in environmental factors. We recorded the temperature, relative humidity and the precipitation for 120 h (Table 1). There was not much change in temperature and the relative humidity values during the study period. However, these parameters may vary every hour during day and night. Thus, further study is needed to determine the association between environmental factors and the survival of bacterial cells on produce surfaces. The bacterial strains used in the studies could have been another factor [54]. Weller et al. (2017) [46] observed a significantly lower die-off rate on the lettuce head samples harvested after a rain event. We did not have sufficient data to establish an association between precipitation and die-off. Our study showed that waiting days between contamination and harvest could be a way to reduce the bacterial load on produce surfaces. However, the die-off rate may be dependent on multiple factors.

**Table 1:** Weather conditions during the study period.

Day (Hour)	Day light length (H:Min:S)	Temperature (^o^C)			Relative humidity (%)		Average Precipitation (Inches)
		Low	High	Day Average	Low	High	
**Day 1** **(0 h)**	NA	24.4	34.4	29.4	48	94	0.93
**Day 2** **(24 h)**	13:49:50	24.4	35.5	30.0	46	91	ND
**Day 3** **(48 h)**	13:48:45	25.0	35.5	30.5	52	90	ND
**Day 4** **(72 h)**	13:47:37	23.8	33.3	28.8	54	90	0.04
**Day 5** **(96 h)**	13:46:28	23.8	35.0	29.4	54	94	ND
**Day 6** **(120 h)**	13:45:18	23.5	32.7	28.8	57	90	ND

ND: Not detected. Temperature: low, high, and day average. Relative humidity: low and high. These weather data were retrieved from https://www.wunderground.com. The temperature values in Fahrenheit were converted to centigrade.

### Attachment strength of *E*. *coli* on watermelon surface increased over time

The ability of bacterial pathogens to attach and form biofilm to produce surfaces is one of the biggest concerns in produce safety. Microorganisms are highly tolerant of various disinfectants when they are in a strongly attached form or in biofilms [55]. In our study, the attachment strength of *E*. *coli* on watermelon surfaces increased with time. The proportion of strongly attached cells to loosely attached cells reversed after 24 h. The majority of survivors or culturable *E*. *coli* cells were in strongly attached form, and the increase in S_R_ value over time was mainly attributed to the decrease in loosely attached population. Therefore, further study is needed to understand the fate of *E*. *coli* cells on produce surfaces, especially die-off and the posibility of transforming into strongly attached cells.Our S_R_ results agree with the findings of other studies, which demonstrated the increasing attachment strength of pathogens on produce (vegetables) surfaces over time [14, 15, 38, 56]. The attachment strength (*S*_*R*_ value) of *E*. *coli* O157: H7 increased from 0.09 to 0.45 on intact cabbage surface after 24 h [38]. In another study, Ells and Hansen [15] reported that the initial S_R_ of *L*. *monocytogenes* to intact cabbage surfaces was 0.66, which increased to 0.82 after 24 h of storage at 37°C.

The initial step of the bacterial colonization to produce surfaces is the reversible attachment [55]. In our study, the proportion of loosely attached cells was significantly higher than strongly attached cells at 0 h, after inoculation. Other studies also reported similar results [14]. This result may be attributed to the initial reversible and weak attachment of bacterial cells. One of the subsequent steps of the colonization involves the production of the exopolymeric substances (EPS) which leads to an irreversible attachment [55]. Bacteria use this mechanism to protect themselves from the harsh environment of phyllosphere and to buffer environmental changes such as nutrient stress, desiccation and UV irradiation [47, 57, 58]. Scanning electron micrograph showed a different bacterial arrangement after 24 h, which may be associated with EPS production. The bacterial cells were enclosed in a structure similar to the extracellular polymeric matrix demonstrated by other studies [15, 47]. Annous et al (2005) [47] observed a fibrillary material after 2 h, and *salmonella* cells embedded in an extracellular polymeric substance in 24 h at 10 & 20^o^ C. Ells and Hansen (2006) [15] observed a greater number of clusters of *Listeria* spp with an extrapolymeric coating (biofilm) on cabbage surfaces after 24 h. These results indicate that the higher level of bacterial attachment might be associated with the production of the exopolymeric substance. If the bacterial cells are embedded in an exopolymeric substance, even the surfactants based washing becomes ineffective in removing them from produce surfaces.

The bacterial attachment and biofilm formation to produce surface are complex processes which may alter physicochemical properties of both bacteria and plant surface [56, 59]. In *E*. *coli*, curli has been found to be associated with the attachment and biofilm formation on biotic and abiotic surfaces [38, 39]. Curli-expressing *E*. *coli* O157:H7 strains showed a higher level of attachment to spinach surfaces compared to the curli-deficient mutants [39].The curli expression of *E*. *coli* is dependent on environmental factors such as temperature, oxygen level and osmolality [60, 61].In our study, all three *E*. *coli* strains exhibited a better curli expression ability at 32°C. While the field temperature during the study period ranged from 23.5°C to 35.5°C. The favorable environmental temperature might have contributed to the attachment of *E*. *coli* cells to watermelon surfaces. However, bacterial cell surface charge and hydrophobicity could be other potential factors associated with the attachment. Patel et al (2010) [14] reported that strong curli-expressing *E*. *coli* O157:H7 showed a higher surface hydrophobicity and a higher level of attachment to the cabbage and iceberg lettuce surfaces than other weak curli expressing strains.

### The efficacy of the chlorine wash reduces with increase in bacterial attachment level

This study showed that the chlorine treatment was effective on watermelon surfaces immediately after the contamination with *E*. *coli*. However, the efficacy of the treatment decreased significantly when treated after 120 h of contamination. Our results concurred with the findings of other studies [56, 62]. Ölmez & Temur, (2010) [62] found that the sanitizer treatments (including chlorine treatment) reduced 99% of total *E*.*coli* and *L*. *monocytogenes* population on lettuce leaves after 6 h of inoculation. However, after 48 h, the treatment reduced only up to 90% of the bacterial population. In an another study, chlorine treatment was less effective when applied after 60 min of the inoculation than 20 to 40 min of the inoculation on the cantaloupe rinds [56]. The results indicate that the efficacy of the chlorine treatment is dependent on the level of bacterial attachment. The reduced efficacy of chlorine against strongly attached cells or cells enclosed in biofilms may be attributed to the limited penetration ability of this sanitizer into exopolymeric substances or biofilm matrix [63]. Bacterial cells embedded within the crevices and fissures of the watermelon rind may be another factor. Cracks and fissures on melon rind surfaces may allow bacterial cells to enter interior tissues [64]; this phenomenon may limit the access of bacterial cells to the applied sanitizer solutions. *E*. *coli* levels on the watermelons harvested at 0 h and at 120 h, before chlorine treatment, was different. Further study is needed to see if there is an effect of the initial bacterial load on the effectiveness of chlorine treatment on watermelon surfaces.

Overall, we calculated an average daily die-off rate of *E*. *coli* on watermelon surfaces in an agriculture setting in a south central location of the United States. The differences in die-off rate from other studies could be due to a number factors, including environmental conditions (Louisiana versus other locations, summer versus other seasons), level of vegetation in the field, type of produce, type of bacterial strains and length of the study. Further studies, therefore, are needed to identify associated factors and their level of influence on the die-off rate, specifically on human pathogens. We observed an increase in attachment level of *E*. *coli* with time in field condition and a lower efficacy of chlorine treatment on the watermelon surfaces 6 days after the inoculation. The results indicated that the efficacy of the chlorine might be dependent on the attachment level of bacterial cells. The results reported in this study may be useful while developing pre-harvest and post-harvest risk management strategies.

## Supporting information

S1 Appendix Natural *E*.*coli* levels on upper half and lower half surfaces of watermelons. (DOCX)

## References

[1] Crowe SJ, Mahon BE, Vieira AR, Gould LH. Vital signs: multistate foodborne outbreaks-United States, 2010–2014. MMWR Morb Mortal Wkly Rep. 2015;64(43):1221–5. Available from: https://www.cdc.gov/mmwr/preview/mmwrhtml/mm6443a4.htm 26540483 10.15585/mmwr.mm6443a4

[2] Herman K, Hall A, Gould L. Outbreaks attributed to fresh leafy vegetables, United States, 1973–2012. Epidemiol. Infect. 2015;143(14):3011–21. 10.1017/S0950268815000047 25697407 PMC4591532

[3] Graça A, Esteves E, Nunes C, Abadias M, Quintas C. Microbiological quality and safety of minimally processed fruits in the marketplace of southern Portugal. Food Control. 2017;73:775–83.

[4] Scharff RL. Health-related costs from foodborne illness in the United States. The Produce Safety Project at Georgetown University. 2010. Available from: http://citeseerx.ist.psu.edu/viewdoc/download?doi=10.1.1.172.4518&rep=rep1&type=pdf [cited 2018].

[5] Ackers M-L, Mahon BE, Leahy E, Goode B, Damrow T, Hayes PS, et al An outbreak of *Escherichia coli* O157: H7 infections associated with leaf lettuce consumption. J. Infect. Dis. 1998;177(6):1588–93. 9607837 10.1086/515323

[6] Leff JW, Fierer N. Bacterial communities associated with the surfaces of fresh fruits and vegetables. PloS one. 2013; 8(3), e59310 10.1371/journal.pone.0059310 23544058 PMC3609859

[7] Gelting RJ, Baloch MA, Zarate-Bermudez MA, Selman C. Irrigation water issues potentially related to the 2006 multistate *E*. *coli* O157: H7 outbreak associated with spinach. Agric Water Manag. 2011;98(9):1395–402.

[8] Park S, Szonyi B, Gautam R, Nightingale K, Anciso J, Ivanek R. Risk factors for microbial contamination in fruits and vegetables at the preharvest level: a systematic review. J. Food Prot. 2012;75(11):2055–81. 10.4315/0362-028X.JFP-12-160 23127717

[9] Olaimat AN, Holley RA. Factors influencing the microbial safety of fresh produce: a review. Food Microbiol. 2012;32(1):1–19. 10.1016/j.fm.2012.04.016 22850369

[10] Atwill ER, Chase JA, Oryang D, Bond RF, Koike ST, Cahn MD, et al Transfer of *Escherichia coli* O157: H7 from simulated wildlife scat onto romaine lettuce during foliar irrigation. Food Prot. 2015;78(2):240–7. 10.4315/0362-028X.JFP-14-277 25710137

[11] Weller DL, Kovac J, Kent DJ, Roof S, Tokman JI, Mudrak E, et al *Escherichia coli* transfer from simulated wildlife feces to lettuce during foliar irrigation: A field study in the Northeastern United States. Food Microbiol. 2017;68:24–33. 10.1016/j.fm.2017.06.009 28800822

[12] Sela S, Nestel D, Pinto R, Nemny-Lavy E, Bar-Joseph M. Mediterranean fruit fly as a potential vector of bacterial pathogens. Appl. Environ. Microbiol. 2005;71(7):4052–6. 10.1128/AEM.71.7.4052-4056.2005 16000820 PMC1169043

[13] Cooley M, Carychao D, Crawford-Miksza L, Jay MT, Myers C, Rose C, et al Incidence and tracking of *Escherichia coli* O157: H7 in a major produce production region in California. PLoS one. 2007;2(11):e1159 10.1371/journal.pone.0001159 18174909 PMC2174234

[14] Patel J, Sharma M. Differences in attachment of *Salmonella enterica* serovars to cabbage and lettuce leaves. Int. J. Food Microbiol. 2010;139(1–2):41–7. 10.1016/j.ijfoodmicro.2010.02.005 20226552

[15] Ells TC, Hansen LT. Strain and growth temperature influence *Listeria* spp. attachment to intact and cut cabbage. Int. J. Food Microbiol. 2006;111(1):34–42. 10.1016/j.ijfoodmicro.2006.04.033 16824634

[16] Toyofuku M, Inaba T, Kiyokawa T, Obana N, Yawata Y, Nomura N. Environmental factors that shape biofilm formation. Biosci. Biotechnol. Biochem. 2016;80(1):7–12. 10.1080/09168451.2015.1058701 26103134

[17] Iturriaga MH, Escartin EF, Beuchat LR, Martinez-Peniche R. Effect of inoculum size, relative humidity, storage temperature, and ripening stage on the attachment of *Salmonella Montevideo* to tomatoes and tomatillos. J. Food Prot. 2003;66(10):1756–61. 14572209 10.4315/0362-028x-66.10.1756

[18] Van Gerven N, Klein RD, Hultgren SJ, Remaut H. Bacterial amyloid formation: structural insights into curli biogensis. Trends Microbiol. 2015;23(11):693–706. 10.1016/j.tim.2015.07.010 26439293 PMC4636965

[19] Alegbeleye OO, Singleton I, Sant’Ana AS. Sources and contamination routes of microbial pathogens to fresh produce during field cultivation: a review. Food Microbiol.2018;73:177–208. 10.1016/j.fm.2018.01.003 29526204 PMC7127387

[20] Morris CE, Monier J-M. The ecological significance of biofilm formation by plant-associated bacteria. Annu Rev Phytopathol. 2003;41(1):429–53. 12730399 10.1146/annurev.phyto.41.022103.134521

[21] Lee WH, Wahman DG, Bishop PL, Pressman JG. Free chlorine and monochloramine application to nitrifying biofilm: comparison of biofilm penetration, activity, and viability. Environ. Sci. Technol. 2011;45(4):1412–9. 10.1021/es1035305 21226531

[22] Ukuku DO, Sapers GM. Effect of sanitizer treatments on *Salmonella Stanley* attached to the surface of cantaloupe and cell transfer to fresh-cut tissues during cutting practices. J. Food Prot. 2001;64(9):1286–91. 11563501 10.4315/0362-028x-64.9.1286

[23] Ryu J-H, Beuchat LR. Biofilm formation by Escherichia coli O157: H7 on stainless steel: effect of exopolysaccharide and curli production on its resistance to chlorine. Appl. Environ. Microbiol. 2005;71(1):247–54. 10.1128/AEM.71.1.247-254.2005 15640194 PMC544232

[24] Kondo N, Murata M, Isshiki K. Efficiency of sodium hypochlorite, fumaric acid, and mild heat in killing native microflora and *Escherichia coli* O157: H7, *Salmonella Typhimurium* DT104, and *Staphylococcus aureus* attached to fresh-cut lettuce. J. Food Prot. 2006;69(2):323–9. 16496572 10.4315/0362-028x-69.2.323

[25] Brandl MT. Fitness of human enteric pathogens on plants and implications for food safety. Annu. Rev. Phytopathol. 2006;44:367–92. 10.1146/annurev.phyto.44.070505.143359 16704355

[26] Moyne A-l Sudarshana MR, Blessington T Koike ST, Cahn MD Harris LJ. Fate of *Escherichia coli* O157: H7 in field-inoculated lettuce. Food Microbiol. 2011;28(8):1417–25. 10.1016/j.fm.2011.02.001 21925023

[27] Bezanson G, Delaquis P, Bach S, McKellar R, Topp E, Gill A, et al Comparative examination of *Escherichia coli* O157: H7 survival on romaine lettuce and in soil at two independent experimental sites. J. Food Prot. 2012;75(3):480–7. 10.4315/0362-028X.JFP-11-306 22410221

[28] Tomás-Callejas A, López-Velasco G, Camacho AB, Artés F, Artés-Hernández F, Suslow TV. Survival and distribution of *Escherichia coli* on diverse fresh-cut baby leafy greens under preharvest through postharvest conditions. Int. J. Food Microbiol. 2011;151(2):216–22. 10.1016/j.ijfoodmicro.2011.08.027 21924789

[29] Nyeleti C, Cogan T, Humphrey T. Effect of sunlight on the survival of *Salmonella* on surfaces. J Appl Microbiol. 2004;97(3):617–20. 10.1111/j.1365-2672.2004.02335.x 15281943

[30] FDA US. FSMA final rule on produce safety. Internet site: http://www.fda.gov/food/guidanceregulation/fsma/ucm334114.htm (Accessed February 2, 2016); 2015.

[31] Gradl JA, Worosz MR. Assessing the Scientific Basis of the Agricultural Water Provision of the FSMA Produce Safety Rule. Food Drug Law J 2017;72:451.

[32] Allende A, Truchado P, Lindqvist R, Jacxsens L. Quantitative Microbial Exposure Modelling as tool to evaluate the impact of contamination level of surface irrigation water and seasonality on fecal hygiene indicator *E*. *coli* in leafy green production. Food Microbiol. 2018;75:82–89. 10.1016/j.fm.2018.01.016 30056967

[33] Walsh KA, Bennett SD, Mahovic M, Gould LH. Outbreaks associated with cantaloupe, watermelon, and honeydew in the United States, 1973–2011. Foodborne Pathog. Dis. 2014;11(12):945–52. 10.1089/fpd.2014.1812 25407556 PMC4627691

[34] Byrne L, Fisher I, Peters T, Mather A, Thomson N, Rosner B, et al A multi-country outbreak of *Salmonella Newport* gastroenteritis in Europe associated with watermelon from Brazil, confirmed by whole genome sequencing: October 2011 to January 2012. Euro Surveill. 2014;19(31):6 25138971 10.2807/1560-7917.es2014.19.31.20866 PMC4516299

[35] Abberton CL, Bereschenko L, van der Wielen PW, Smith CJ. Survival, biofilm formation, and growth potential of environmental and enteric *Escherichia coli* strains in drinking water microcosms. Appl. Environ. Microbiol. 2016;82(17):5320–31. 10.1128/AEM.01569-16 27342552 PMC4988207

[36] Harris LJ, Bender J, Bihn EA, Blessington T, Danyluk MD, Delaquis P, et al A framework for developing research protocols for evaluation of microbial hazards and controls during production that pertain to the quality of agricultural water contacting fresh produce that may be consumed raw. J. Food Prot. 2012;75(12):2251–73. Epub 2012/12/06. 10.4315/0362-028X.JFP-12-252 . 23212026

[37] Adhikari A, Bary A, Cogger C, James C, Ünlü G, Killinger K. Thermal and Starvation Stress Response of *Escherichia coli* O157: H7 Isolates Selected from Agricultural Environments. J. Food Prot. 2016;79(10):1673–9. 10.4315/0362-028X.JFP-16-115 28221847

[38] Patel J, Sharma M, Ravishakar S. Effect of curli expression and hydrophobicity of *Escherichia coli* O157: H7 on attachment to fresh produce surfaces. J Appl Microbiol. 2011;110(3):737–45. 10.1111/j.1365-2672.2010.04933.x 21205101

[39] Macarisin D, Patel J, Bauchan G, Giron JA, Sharma VK. Role of curli and cellulose expression in adherence of *Escherichia coli* O157: H7 to spinach leaves. Foodborne Pathog. Dis. 2012;9(2):160–7. 10.1089/fpd.2011.1020 22315954

[40] Parnell TL, Harris LJ, Suslow TV. Reducing *Salmonella* on cantaloupes and honeydew melons using wash practices applicable to postharvest handling, foodservice, and consumer preparation. Int. J. Food Microbiol. 2005;99(1):59–70. 10.1016/j.ijfoodmicro.2004.07.014 15718029

[41] Fleming P, Pool B, Fruit U. Commodity specific food safety guidelines for the melon supply chain. Produce Marketing Association. 2005:1–39.

[42] Chhetri VS, Fontenot K, Strahan R, Yemmireddy VK, Parraga Estrada KJ, Adhikari A. Effect of surrounding vegetation on microbial survival or die‐off on watermelon surface in an agriculture setting. J Food Saf. 2018:e12520.

[43] Yu K, Newman MC, Archbold DD, Hamilton-Kemp TR. Survival of *Escherichia coli* O157: H7 on strawberry fruit and reduction of the pathogen population by chemical agents. J. Food Prot. 2001;64(9):1334–40. 11563509 10.4315/0362-028x-64.9.1334

[44] Barker-Reid F, Harapas D, Engleitner S, Kreidl S, Holmes R, Faggian R. Persistence of *Escherichia coli* on injured iceberg lettuce in the field, overhead irrigated with contaminated water. J. Food Prot. 2009;72(3):458–64. 19343931 10.4315/0362-028x-72.3.458

[45] Wood J, Bezanson G, Gordon R, Jamieson R. Population dynamics of *Escherichia coli* inoculated by irrigation into the phyllosphere of spinach grown under commercial production conditions. Int. J. Food Microbiol. 2010;143(3):198–204. 10.1016/j.ijfoodmicro.2010.08.022 20864201

[46] Weller DL, Kovac J, Roof S, Kent DJ, Tokman JI, Kowalcyk B, et al Survival of *Escherichia coli* on lettuce under field conditions encountered in the northeastern United States. J. Food Prot. 2017;80(7):1214–21. 10.4315/0362-028X.JFP-16-419 28632416

[47] Annous BA, Solomon EB, Cooke PH, Burke A. Biofilm formation by *Salmonella* spp. on cantaloupe melons. J Food Saf. 2005;25(4):276–87.

[48] De Carvalho CC. Biofilms: recent developments on an old battle. Recent patents on biotechnology. 2007;1(1):49–57. 19075832 10.2174/187220807779813965

[49] Beuchat LR. Ecological factors influencing survival and growth of human pathogens on raw fruits and vegetables. Microbes Infect. 2002;4(4):413–23. 11932192 10.1016/s1286-4579(02)01555-1

[50] Brooks AN, Turkarslan S, Beer KD, Yin Lo F, Baliga NS. Adaptation of cells to new environments. Wiley Interdiscip Rev Syst Biol Med. 2011;3(5):544–61. 10.1002/wsbm.136 21197660 PMC3081528

[51] Phaiboun A, Zhang Y, Park B, Kim M. Survival kinetics of starving bacteria is biphasic and density-dependent. PLoS Comput. Biol. 2015;11(4):e1004198 10.1371/journal.pcbi.1004198 25838110 PMC4383377

[52] Hutchison M, Avery S, Monaghan J. The air‐borne distribution of zoonotic agents from livestock waste spreading and microbiological risk to fresh produce from contaminated irrigation sources. J Appl Microbiol. 2008;105(3):848–57. 10.1111/j.1365-2672.2008.03811.x 18422957

[53] Xu A, Buchanan RL, Micallef SA. Impact of mulches and growing season on indicator bacteria survival during lettuce cultivation Int. J. Food Microbiol. 2016;224:28–39. 10.1016/j.ijfoodmicro.2016.02.013 26938806

[54] Stine SW, Song I, Choi CY, Gerba CP. Effect of relative humidity on preharvest survival of bacterial and viral pathogens on the surface of cantaloupe, lettuce, and bell peppers. J. Food Prot. 2005;68(7):1352–8. 16013370 10.4315/0362-028x-68.7.1352

[55] Jahid IK, Ha S-D. A review of microbial biofilms of produce: future challenge to food safety. Food Sci. Biotechnol. 2012;21(2):299–316.

[56] Ukuku DO, Fett WF. Relationship of cell surface charge and hydrophobicity to strength of attachment of bacteria to cantaloupe rind. J. Food Prot. 2002;65(7):1093–9. 12117240 10.4315/0362-028x-65.7.1093

[57] Monier J-M, Lindow S. Aggregates of resident bacteria facilitate survival of immigrant bacteria on leaf surfaces. Microbial Ecology. 2005;49(3):343–52. 10.1007/s00248-004-0007-9 16003469

[58] Elasri MO, Miller RV. Study of the response of a biofilm bacterial community to UV radiation. Appl. Environ. Microbiol.1999;65(5):2025–31. 10223995 10.1128/aem.65.5.2025-2031.1999 PMC91292

[59] Hirano SS, Upper CD. Bacteria in the Leaf Ecosystem with Emphasis on *Pseudomonas syringae*—a Pathogen, Ice Nucleus, and Epiphyte. Microbiol Mol Biol Rev. 2000;64(3):624–53. 10974129 10.1128/mmbr.64.3.624-653.2000 PMC99007

[60] Boyer RR, Sumner SS, Williams RC, Pierson MD, Popham DL, Kniel KE. Influence of curli expression by *Escherichia coli* O157: H7 on the cell's overall hydrophobicity, charge, and ability to attach to lettuce. J. Food Prot. 2007;70(6):1339–45. 17612061 10.4315/0362-028x-70.6.1339

[61] Evans ML, Chapman MR. Curli biogenesis: order out of disorder. Biochim. Biophys. Acta. 2014;1843(8):1551–8. 10.1016/j.bbamcr.2013.09.010 24080089 PMC4243835

[62] Ölmez H, Temur S. Effects of different sanitizing treatments on biofilms and attachment of *Escherichia coli* and *Listeria monocytogenes* on green leaf lettuce. LWT—Food Sci. Technol. 2010;43(6):964–70.

[63] De Beer D, Stoodley P, Roe F, Lewandowski Z. Effects of biofilm structures on oxygen distribution and mass transport. Biotechnol. Bioeng. 1994;43(11):1131–8. 10.1002/bit.260431118 18615526

[64] Gautam D, Dobhal S, Payton ME, Fletcher J, Ma LM. Surface Survival and Internalization of *Salmonella* through Natural Cracks on Developing Cantaloupe Fruits, Alone or in the Presence of the Melon Wilt Pathogen *Erwinia tracheiphila*. PloS one. 2014;9(8):e105248 10.1371/journal.pone.0105248 25147942 PMC4141780

