# Supplementary material for: Attachment strength and on-farm die-off rate of Escherichia coli on watermelon surfaces
Source: PLoS One. 2019 Jan 8;14(1):e0210115. doi: 10.1371/journal.pone.0210115 (PMC6324798; doi:10.1371/journal.pone.0210115)
Supplement: S1 Appendix — (DOCX) [file pone.0210115.s001.docx]

**S1 Appendix**: Natural *E.coli* levels on upper half and lower half surfaces of watermelons

| Sample code | Location on the watermelons surfaces | Total count CFU/sample | Surface Area of the samples (cm^2^) | Count CFU/cm^2^ | Count log CFU/cm^2^ |
| --- | --- | --- | --- | --- | --- |
| 103 (a) | Upper half | 2000 | 215.60 | 9.28 | 0.97 |
|  | Lower half | 0 | 215.60 | 0.00 | #NUM! |
| 101 (a) | Upper half | 600 | 190.62 | 3.15 | 0.50 |
|  | Lower half | 0 | 190.62 | 0.00 | #NUM! |
| 101 (b) | Upper half | 0 | 285.10 | 0.00 | #NUM! |
|  | Lower half | 6000 | 285.10 | 21.05 | 1.32 |
| 101 (c) | Upper half | 0 | 227.00 | 0.00 | #NUM! |
|  | Lower half | 2000 | 227.00 | 8.81 | 0.94 |
| 202 (a) | Upper half | 100 | 217.58 | 0.46 | -0.34 |
|  | Lower half | 0 | 217.58 | 0.00 | #NUM! |
| 102 (a) | Upper half | 0 | 263.39 | 0.00 | #NUM! |
|  | Lower half | 0 | 263.39 | 0.00 | #NUM! |
| 102 (b) | Upper half | 0 | 268.15 | 0.00 | #NUM! |
|  | Lower half | 0 | 268.15 | 0.00 | #NUM! |
| 105 | Upper half | 0 | 304.02 | 0.00 | #NUM! |
|  | Lower half | 0 | 304.02 | 0.00 | #NUM! |
| 104 | Upper half | 0 | 283.15 | 0.00 | #NUM! |
|  | Lower half | 0 | 283.15 | 0.00 | #NUM! |
| 202 (b) | Upper half | 0 | 306.63 | 0.00 | #NUM! |
|  | Lower half | 4666.67 | 306.63 | 15.22 | 1.18 |
| 203 | Upper half | 0 | 230.96 | 0.00 | #NUM! |
|  | Lower half | 0 | 230.96 | 0.00 | #NUM! |
| 205 (a) | Upper half | 100 | 229.03 | 0.44 | -0.36 |
|  | Lower half | 100000 | 229.03 | 436.62 | 2.64 |
| 206 | Upper half | 0 | 273.62 | 0.00 | #NUM! |
|  | Lower half | 0 | 273.62 | 0.00 | #NUM! |
| 301 | Upper half | 0 | 222.29 | 0.00 | #NUM! |
|  | Lower half | 0 | 222.29 | 0.00 | #NUM! |
| 303 (a) | Upper half | 0 | 291.36 | 0.00 | #NUM! |
|  | Lower half | 0 | 291.36 | 0.00 | #NUM! |
| 303 (b) | Upper half | 0 | 197.62 | 0.00 | #NUM! |
|  | Lower half | 0 | 197.62 | 0.00 | #NUM! |
| 304 | Upper half | 0 | 273.62 | 0.00 | #NUM! |
|  | Lower half | 0 | 273.62 | 0.00 | #NUM! |
| 305 (a) | Upper half | 0 | 275.09 | 0.00 | #NUM! |
|  | Lower half | 20000 | 275.09 | 72.70 | 1.86 |
| 305 (b) | Upper half | 0 | 299.16 | 0.00 | #NUM! |
|  | Lower half | 0 | 299.16 | 0.00 | #NUM! |
| 305 (c) | Upper half | 0 | 245.15 | 0.00 | #NUM! |
|  | Lower half | 0 | 245.15 | 0.00 | #NUM! |
| 306 (a) | Upper half | 0 | 288.75 | 0.00 | #NUM! |
|  | Lower half | 0 | 288.75 | 0.00 | #NUM! |
| 306 (b) | Upper half | 0 | 187.89 | 0.00 | #NUM! |
|  | Lower half | 0 | 187.89 | 0.00 | #NUM! |
| 103 (b) | Upper half | 100 | 269.19 | 0.37 | -0.43 |
|  | Lower half | 0 | 269.19 | 0.00 | #NUM! |
| 205 (b) | Upper half | 0 | 226.45 | 0.00 | #NUM! |
|  | Lower half | 55000 | 226.45 | 242.88 | 2.39 |
| 205 (c) | Upper half | 0 | 224.45 | 0.00 | #NUM! |
|  | Lower half | 0 | 227.45 | 0.oo | #NUM! |
